# Insula reactivity mediates subjective isolation stress in alexithymia

**DOI:** 10.1038/s41598-021-94799-w

**Published:** 2021-07-28

**Authors:** Mitjan Morr, Jana Lieberz, Michael Dobbelstein, Alexandra Philipsen, René Hurlemann, Dirk Scheele

**Affiliations:** 1 Division of Medical Psychology, Department of Psychiatry and Psychotherapy, University Hospital Bonn, Venusberg-Campus 1, 53127 Bonn, Germany; 2 Department of Psychiatry and Psychotherapy, University Hospital Bonn, 53127 Bonn, Germany; 3 Department of Psychiatry, School of Medicine and Health Sciences, University of Oldenburg, Hermann-Ehlers-Str. 7, 26129 Oldenburg, Germany; 4 Research Center Neurosensory Science, University of Oldenburg, 26129 Oldenburg, Germany

**Keywords:** Neuroscience, Psychology, Risk factors

## Abstract

The risk for developing stress-related disorders is elevated in individuals with high alexithymia, a personality trait characterized by impaired emotional awareness and interpersonal relating. However, it is still unclear how alexithymia alters perceived psychosocial stress and which neurobiological substrates are mechanistically involved. To address this question, we examined freshmen during transition to university, given that this period entails psychosocial stress and frequently initiates psychopathology. Specifically, we used a functional magnetic resonance imaging emotional face matching task to probe emotional processing in 54 participants (39 women) at the beginning of the first year at university and 6 months later. Furthermore, we assessed alexithymia and monitored perceived psychosocial stress and loneliness via questionnaires for six consecutive months. Perceived psychosocial stress significantly increased over time and initial alexithymia predicted subjective stress experiences via enhanced loneliness. On the neural level, alexithymia was associated with lowered amygdala responses to emotional faces, while loneliness correlated with diminished reactivity in the anterior insular and anterior cingulate cortex. Furthermore, insula activity mediated the association between alexithymia and loneliness that predicted perceived psychosocial stress. Our findings are consistent with the notion that alexithymia exacerbates subjective stress via blunted insula reactivity and increased perception of social isolation.

## Introduction

Major life events such as the transition from school to university or from work to retirement involve changes in the social environment and are frequently accompanied by increased psychosocial stress^1^. The allostatic load^2^, that is the wear and tear resulting from chronic overactivity of stress systems, can increase the risk of stress-related disorders like major depression or anxiety. Both environmental factors and interindividual differences modulate the allostatic load. Specifically, the ability to effectively cope with a life stressor is decreased in individuals with high alexithymia^3^, a personality trait characterized by impaired emotional awareness and interpersonal relating. According to the stress-alexithymia hypothesis^4^, the lack of emotional awareness hinders the identification of an event as stressful and the resulting ineffective coping aggravates the allostatic load. In fact, there is accumulating evidence that alexithymia has detrimental effects on mental and physical health^5–7^. In addition, alexithymia is associated with dysfunctional interpersonal bonding, which might lead to distressful feelings of loneliness if the quality or quantity of social relationships does not satisfy a person’s need to belong^8,9^. Loneliness and social withdrawal in turn foster depressive symptomatology^10^ and may increase the risk of relapse^11^. Furthermore, recent studies support close associations between loneliness, atypical physiological responses to acute stress and detrimental emotion-oriented stress coping strategies^12–15^. Collectively, not only the objective availability of support via social networks may modulate the allostatic load during transition phases, but also the subjective perception of social connectedness. Therefore, alexithymia might negatively impact psychological well-being and mental health via impaired interpersonal relating^16,17^. However, while the stress-alexithymia hypothesis is well established, it is still unclear whether alexithymia affects perceived stress during social transition phases by enhancing feelings of loneliness. Moreover, little is known about the underlying neurobiological mechanisms that promote the detrimental effects of alexithymia on stress responses.

Importantly, alexithymia and loneliness seem to affect similar neural pathways: a meta-analysis of neuroimaging studies^18^ revealed that high levels of alexithymia are associated with blunted responses to emotional stimuli (e.g. happy and fearful faces) in a limbic neurocircuitry including the amygdala and insular cortex and elevated responses in the anterior cingulate cortex (ACC) that may reflect difficulties in identifying and regulating emotions. Likewise, in highly lonely individuals, pleasant social stimuli elicited less activity in the striatum, amygdala, insula and ACC^19^. Of note, these brain regions have been identified as important neural hubs of stress resilience such that robust amygdala responses to emotional stimuli and functional coupling of ACC-insula circuitry might promote adaptive stress responses^20,21^. Moreover, a recent study demonstrated that targeted amygdala neurofeedback improves stress coping and reduces alexithymia^22^, further strengthening the assumption that alexithymia and loneliness prevent favorable stress response by shared neural response patterns.

The current study thus aims to probe whether alexithymia might affect perceived stress by enhancing feelings of loneliness and to examine which neural substrates are involved. Therefore, we measured alexithymic traits and neural activation patterns in response to social stimuli (emotional faces) during functional magnetic resonance imaging (fMRI) in a sample consisting of 54 healthy freshmen. Participants were monitored during their first 6 months of the transition to university. Each month, participants completed questionnaires measuring their loneliness and their subjective stress experiences during this major life event. Specifically, we hypothesized a positive correlation between alexithymia and subjective stress response across time and that this relationship would be mediated by feelings of loneliness. Given the intertwined phenotype of alexithymia and loneliness as well as the overlapping neural correlates of both constructs, we predicted that both higher alexithymic traits and higher loneliness levels would be associated with altered responses to emotional face stimuli in the ACC, insula and amygdala. To this end, we used alexithymia and loneliness scores as continuous covariates in the analyses. Finally, we expected that the link between alexithymia and perceived stress would be mechanistically mediated by altered activity in these brain regions.

## Results

### Behavioral results

**Figure 1:**
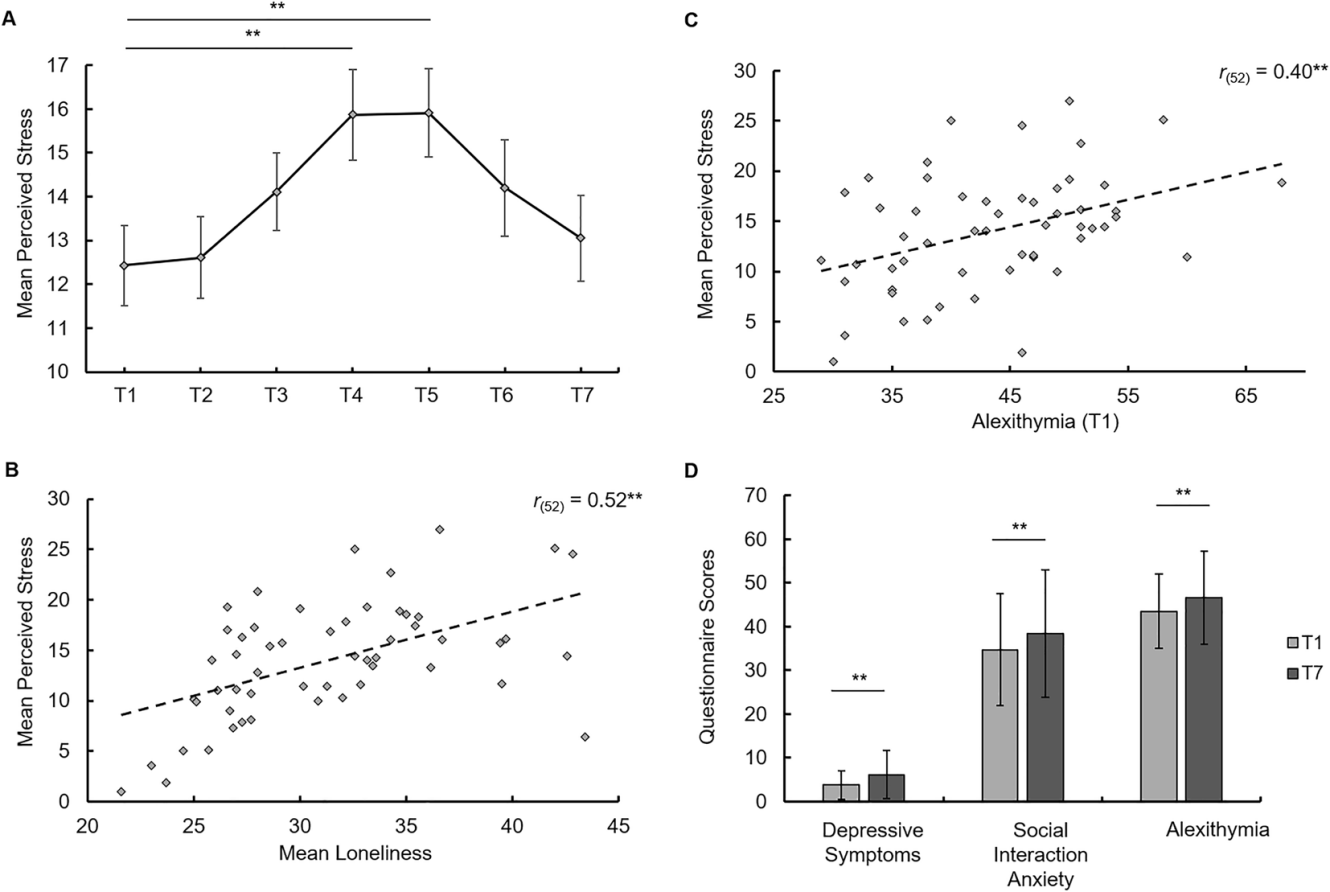
Perceived stress significantly changed over time and peaked in months four and five of the observation period (**A**). Mean perceived stress positively correlated with mean loneliness (**B**) and alexithymia (**C**) at study entry. Depressive symptoms, social interaction anxiety and alexithymia significantly increased after 6 months (**D**). Error bars show the standard error of the mean. **p < 0.01, T1–T7, first to seventh month.

Stress levels changed significantly over time (*F*_(6,294)_ = 4.56, *p* < 0.01, η_p_^2^ = 0.09) and peaked in month four (*t*_(52)_ = 4.48, Bonferroni-corrected *p* (*p*_cor_) < 0.01, *d* = 0.47) and five (*t*_(53)_ = 3.92, *p*_cor_ = 0.02, *d* = 0.49) of the observation period in comparison to the stress levels at study entry (see Fig. 1A), reflecting the first examination phase. In contrast, social network size (*F*_(6,282)_ = 0.96, *p* = 0.43, η_p_^2^ = 0.02) and loneliness scores (*F*_(6,288)_ = 1.69, *p* = 0.14, η_p_^2^ = 0.03) did not significantly change during the time course (see Table S1). As predicted, both the average loneliness (*r*_(52)_ = 0.52, *p* < 0.01; see Fig. 1B) and alexithymia in the first month (T1) positively correlated with the average perceived stress in the 6 months (*r*_(52)_ = 0.40, *p* < 0.01; see Fig. 1C), showing that individuals with greater dysfunctional emotional awareness and higher subjective lack of social connection experienced more stress during the transition phase. In addition, T1 alexithymia positively correlated with psychosocial stress (*r*_(52)_ = 0.49, *p* < 0.01) already at study entry, but was not significantly associated with the increase in stress levels (i.e. maximum stress minus baseline) during the first examination phase (*p* > 0.05), indicating that alexithymia is associated with consistently increased perceived stress levels rather than increased acute stress responsiveness. Furthermore, depressive symptoms (*t*_(53)_ = 3.19, *p* < 0.01, *d* = 0.53), social interaction anxiety (*t*_(53)_ = 3.05 *p* < 0.01 *d* = 0.26) and alexithymia (*t*_(53)_ = 2.83, *p* < 0.01, *d* = 0.32) significantly increased after 6 months (see Fig. 1D).

### fMRI task effects

Across both fMRI sessions, the participants exhibited increased responses to emotional faces (fearful and happy) compared to neutral ones in middle temporal regions (L (left): x, y, z coordinates of peak voxel in Montreal Neurological Institute space (MNI_xyz_): − 60, − 56, 2, *k*_E_ = 125, after familywise error corrections (*p*_FWE_) on cluster level *p*_FWE_ = 0.02; R (right): MNI_xyz_: 58, − 58, 12, *k*_E_ = 198, *p*_FWE_ < 0.01), the inferior temporal gyrus (MNI_xyz_: − 42, − 42, − 16, *k*_E_ = 135, *p*_FWE_ = 0.01) and middle occipital regions (L: MNI_xyz_: − 22, − 98, 0, *k*_E_ = 723, *p*_FWE_ < 0.01; R: MNI_xyz_: 26, − 90, 0, *k*_E_ = 925, *p*_FWE_ < 0.01). Furthermore, subjects showed stronger activation in response to fearful faces relative to neutral faces in clusters including the middle temporal gyrus (L: MNI_xyz_: − 58, − 52, 4, *k*_E_ = 293, *p*_FWE_ < 0.01; R: MNI_xyz_: 58, − 58, 14, *k*_E_ = 533, *p*_FWE_ < 0.01), the left inferior temporal gyrus (MNI_xyz_: − 42, − 44, − 16, *k*_E_ = 206, *p*_FWE_ < 0.01), the right occipital region (MNI_xyz_: 26, − 90, 0, *k*_E_ = 589, *p*_FWE_ < 0.01) and lingual areas in the left hemisphere (MNI_xyz_: − 20, − 90, − 14, *k*_E_ = 591, *p*_FWE_ < 0.01). Moreover, subjects showed increased activity in middle occipital regions (L: MNI_xyz_: − 20, − 98, 2, *k*_E_ = 437, *p*_FWE_ < 0.01; R: MNI_xyz_: 32, − 92, 6, *k*_E_ = 537, *p*_FWE_ < 0.01) in response to happy faces compared to neutral ones. There were no significant whole-brain differences between the first (T1) and the seventh month (T7).

### Correlation analyses of alexithymia and loneliness with brain activation

**Figure 2:**
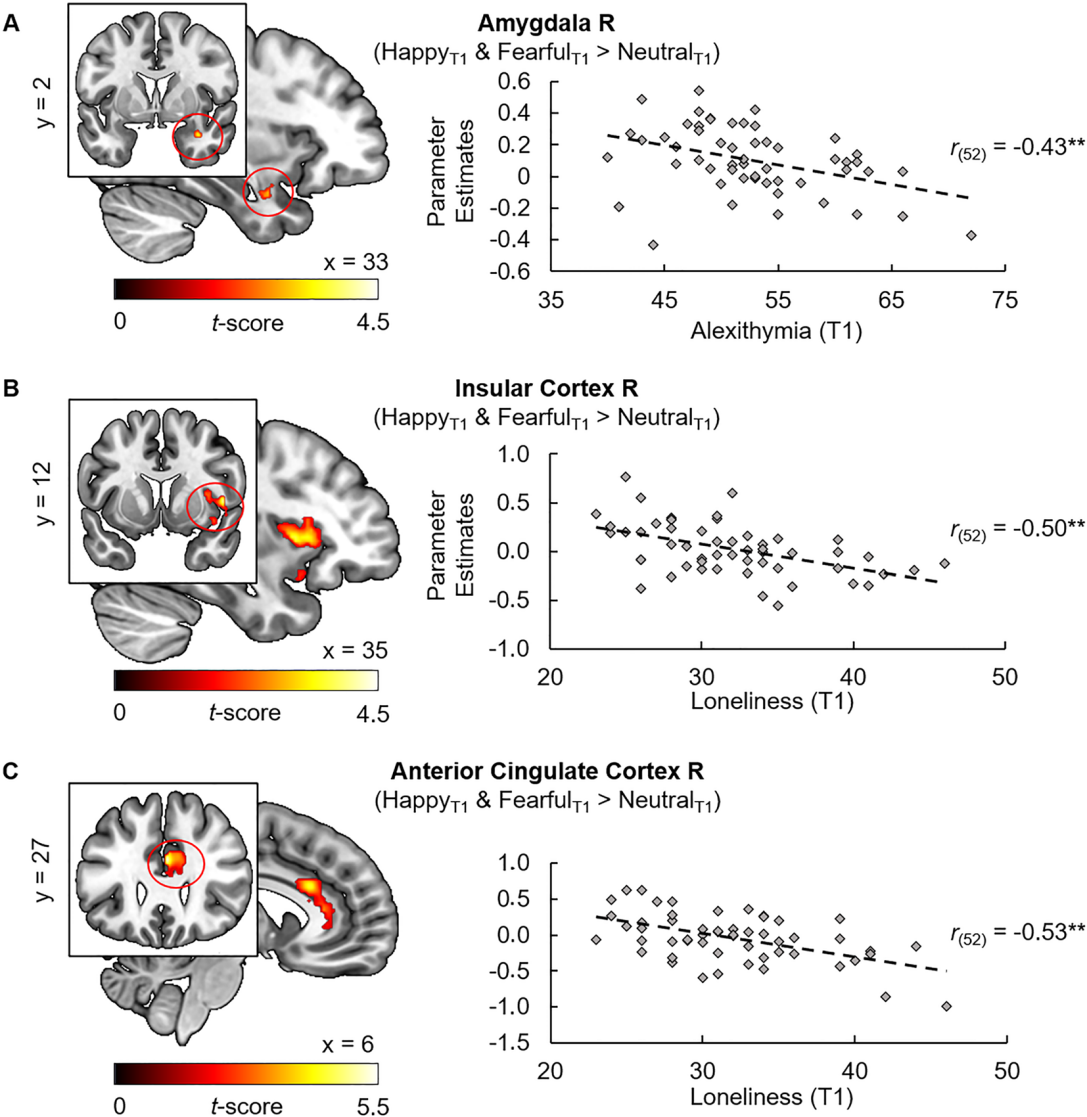
Participants with high alexithymia showed reduced activation to emotional faces compared to neural faces in the right amygdala (**A**; MNI_xyz_: 34, 2, − 24, *t*_(53)_ = 3.55, *p*_FWE_ = 0.03). Individuals with high loneliness exhibited lower responses to emotional faces in the right anterior insular cortex (**B**; MNI_xyz_: 48, 8, 4, *t*_(53)_ = 4.21, *p*_FWE_ = 0.03) and the right anterior cingulate cortex (**C**; MNI_xyz_: 2, 26, 24, *t*_(53)_ = 4.82, *p*_FWE_ < 0.01). For illustration purpose clusters are shown with significance level of *p* = 0.05. **p < 0.01, *FWE* familywise error corrected, *L* left, *MNI* Montreal Neurological Institute, *R* right, *T1* study entry.

Individuals with high alexithymia showed decreased right amygdala responses to emotional faces in contrast to neutral faces at T1 (MNI_xyz_: 34, 2, − 24, *t*_(53)_ = 3.55, *p*_FWE_ = 0.03 on peak level; see Fig. 2A). Furthermore, subjects with higher loneliness exhibited reduced activation in response to emotional faces in the left and right anterior insula (L: MNI_xyz_: − 36, 12, 8, *t*_(53)_ = 4.36, *p*_FWE_ = 0.02; R: MNI_xyz_: 48, 8, 4, *t*_(53)_ = 4.21, *p*_FWE_ = 0.03; see Fig. 2B), and ACC (L: MNI_xyz_: 0, 28, 24, *t*_(53)_ = 4.85, *p*_FWE_ < 0.01; R: MNI_xyz_: 2, 26, 24, *t*_(53)_ = 4.82, *p*_FWE_ < 0.01; see Fig. 2C) at T1. Likewise, loneliness negatively correlated with responses to fearful faces in the left anterior insular cortex (MNI_xyz_: − 34, 10, 10, *t*_(53)_ = 4.73, *p*_FWE_ = 0.01) and ACC (L: MNI_xyz_: 0, 8, 26, *t*_(53)_ = 4.79, *p*_FWE_ = 0.01; MNI_xyz_: 0, 28, 24, *t*_(53)_ = 4.70, *p*_FWE_ = 0.01; R: MNI_xyz_: 2, 26, 24, *t*_(53)_ = 4.52, *p*_FWE_ = 0.01; MNI_xyz_: 2, 8, 28, *t*_(53)_ = 4.03, *p*_FWE_ = 0.03) and anterior insula responses (MNI_xyz_: 34, 12, 4, *t*_(53)_ = 4.12, *p*_FWE_ = 0.04) to happy faces. These associations were not evident at T7.

### Mediation analysis

**Figure 3:**
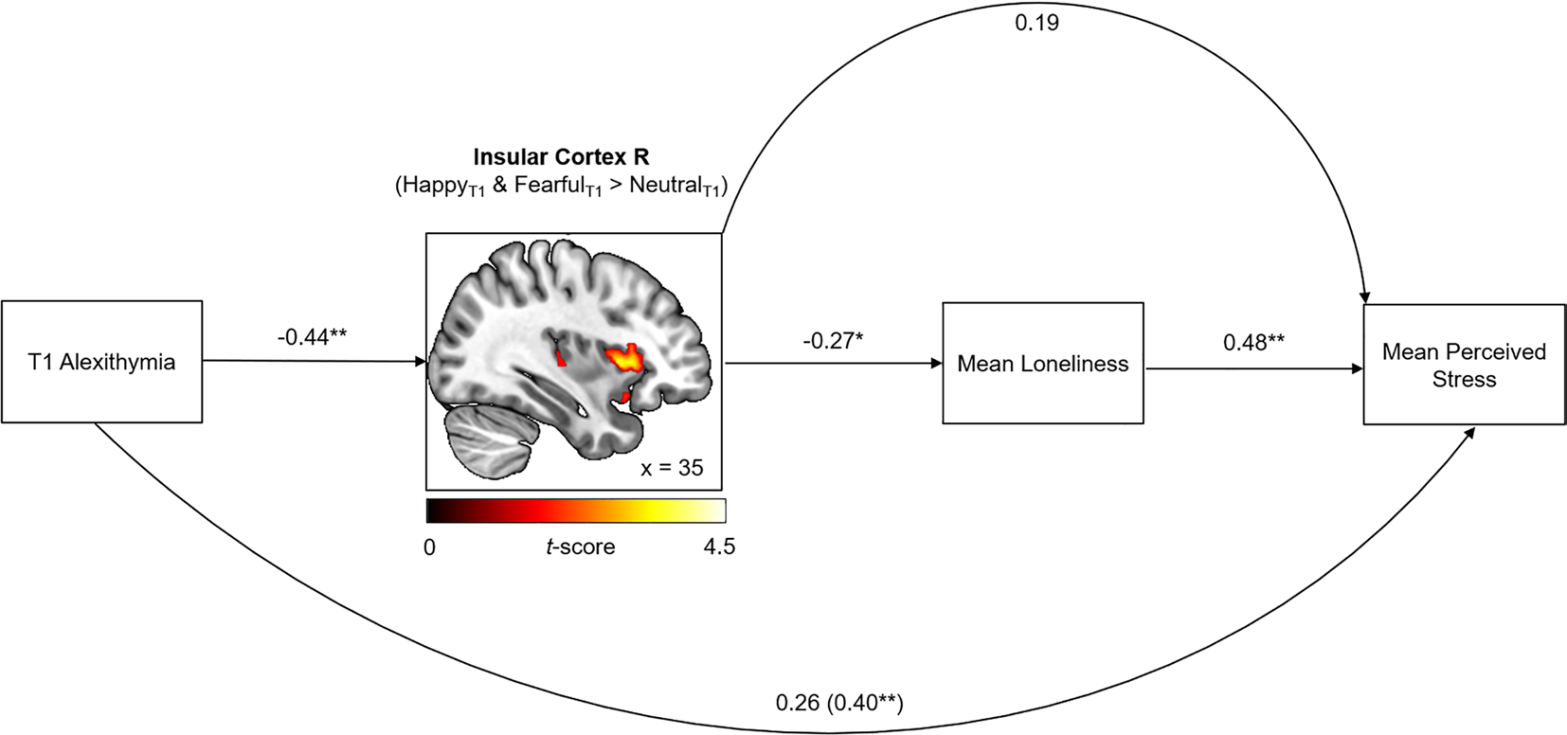
Mean loneliness mediated the relationship between alexithymia at study entry and mean perceived stress ratings. Furthermore, activation of the right insula in response to emotional stimuli at study entry mediated the link between alexithymia and loneliness. Numbers show standardized β coefficients. The β coefficient in brackets shows the total effect without mediators. Insula coordinates are shown in Montreal Neurological Institute space. For illustration purpose, the cluster is shown with a significance level of *p* = 0.05. *p < 0.05, **p < 0.01, *T1* study entry, *R* right.

To examine whether higher levels of alexithymia predicted perceived stress levels by enhancing feelings of loneliness, a first mediation analysis was calculated with alexithymia as predictor for subjective stress and loneliness as mediator. A significant mediation via loneliness [indirect effect of alexithymia on stress via loneliness: β = 0.20, standard error (SE) = 0.10, 95% confidence interval (CI) 0.04–0.43] indicated that the detrimental effects of alexithymia on perceived stress were indeed mediated by loneliness with the direct effect of alexithymia on stress being diminished after including loneliness (total effect of alexithymia on stress: β = 0.40, *p* = 0.003, SE = 0.13, 95% CI 0.15–0.66; direct effect of alexithymia on stress after including loneliness as mediator: β = 0.20, *p* = 0.14, SE = 0.13, 95% CI −0.07 to 0.47). In a second step, we added the parameter estimates of the right amygdala, right ACC and right anterior insula as further mediator variables to the model to elucidate the underlying neural mechanisms. For each brain region, two models were calculated to test potential mediation effects on all pathways (i.e., both serial and parallel mediation effects were tested). The analyses revealed that the link between alexithymia and loneliness was driven by reduced insula reactivity, leading to a significant indirect effect of alexithymia on stress via insula reactivity and loneliness (serial mediation: β = 0.06, SE = 0.04, 95% CI 0.01–0.15, see Fig. 3). Specifically, alexithymia predicted the reduced anterior insula reactivity which was linked to enhanced feelings of loneliness which in turn, predicted subjective stress. This mediation was mainly driven by the Toronto Alexithymia Scale (TAS) factors “difficulties describing feelings” (DDF) and “difficulties identifying feelings” (DIF) (see SI Results). No further mediation effects were observed for the insula, amygdala or ACC (all 95% CIs of further indirect effects via brain activation included zero).

## Discussion

The present study aimed at elucidating the neural mechanisms moderating the link between alexithymic traits, loneliness and stress reactivity during the transition to university. Our results confirmed that loneliness mediated the noxious association between alexithymia and subjective stress during the first 6 months of university. Moreover, we found that the anterior insula plays a crucial role in this process, by mediating the link between alexithymia and loneliness.

Our results provide further support for and extend the stress-alexithymia hypothesis^4^. We were able to replicate previous models suggesting a close link between alexithymia and loneliness^11^ and found that individuals with high alexithymia, especially with difficulties in describing and identifying emotions, experience more stress during transition phases partly because they perceive more subjective social isolation. This finding is consistent with previous studies reporting significant associations between the TAS DIF and DDF subscales and loneliness^23^ as well as a relationship between the TAS DIF subscale and poor adjustment during transition to university and perceived stress^24^. Intriguingly, our results indicate that this mechanism may be driven by diminished insula responses to emotional signals which directly link alexithymia with loneliness. The insular cortex is a hub for interoceptive processing and conscious affect^25^ and endotoxin-induced changes in the glucose metabolism of the right insula positively correlate with changes in social interest^26^. Likewise, individuals with high loneliness have been found to exhibit reduced insula responses during interpersonal trust decisions^27^. Moreover, multiple lines of evidence indicate that insula pathology leads to alexithymia. For instance, dopamine D2-type receptor availability in the insula has been linked to higher alexithymia^28^, the gray matter volume of the insular cortex inversely correlated with alexithymia^29^ and the extent of damage to the anterior insula predicted alexithymia in lesion patients^30^. It has been theorized that insula dysfunction in alexithymia may reflect a transdiagnostic marker of empathic deficits^31^ and our findings in healthy participants point to an additional mechanism such that the dampened insula responses to external emotional cues underlie the association of alexithymia with enhanced perceived social isolation. Along these lines, the observed pattern of results is consistent with the notion that social connectedness requires the ability to flexibly shift between interoceptive and exteroceptive attention^32^ which may be based on recruitment of the anterior insula.

Furthermore, consistent with previous fMRI studies^18,19^, we found decreased amygdala and ACC responses in individuals with high alexithymia and loneliness, respectively. The amygdala has often been linked to alexithymia^18,33^ and a recent study showed that neurofeedback targeting the amygdala during military training not only enhanced stress coping but also decreased alexithymia^22^. Moreover, the amygdala has also been linked to loneliness and social support. For example, a decrease in perceived stress and loneliness was moderated by amygdala volumes^34^ and the experience of social support was regulated by amygdala activity^35^. Likewise, the ACC has been previously linked not only to loneliness but also to alexithymia: ACC size correlates with alexithymia ratings especially in men^36^ and high levels of alexithymia are associated with elevated responses to emotional stimuli in the ACC^18^. Furthermore, the ACC plays a role in social pain processing during social support^37^ and overall seems to be a hub for the integration of social information and empathy^38^. Bearing in mind that neither ACC nor amygdala reactivity mediated effects of alexithymia, the insular cortex seems to be a crucial neural processing hub for the interplay between loneliness and alexithymia. Therefore, neurofeedback training targeting insula activation could lead not only to reduced feelings of loneliness but also to reduced psychosocial stress^22^. In contrast to loneliness, objective social network indices were not significantly associated with alexithymia or perceived stress. Given that in a previous study with college freshmen^39^ psychological stress selectively mediated the association between antibody response to the influenza immunization and loneliness, but not social network size and immunization response, our data provide further support for the notion that the subjective perception of social connectedness may be a more important predictor for stress reactivity during transition phases than the objectively available social contacts.

Interestingly, the trait-dependent reactivity was no longer evident in the second fMRI session 6 months later, indicating either repetition effects and reduced retest-reliability or that a disrupted plasticity as observed in the prefrontal cortex with an attention-shifting task following long-term psychosocial stress^40^ is more pronounced for limbic reactivity to emotional stimuli. Furthermore, we observed an increase in alexithymia scores, potentially elicited by the prolonged subjective stress, which might reflect an acquired secondary alexithymia^41^. As such, these experience-based changes may have masked genuine trait associations in the second fMRI session. Of note, the allostatic load of the transition to university caused a significant increase in depressive symptoms, social interaction anxiety and autistic-like traits after 6 months, thus illustrating that individuals with high alexithymia and loneliness might be at risk not only for poor academic performance but also stress-related psychological disorders due to chronically increased stress levels.

Collectively, our results provide evidence for a close interplay between emotional awareness and perceived social isolation, with dampened insula reactivity serving as a potential underlying mechanism linking alexithymia with loneliness and thus exacerbating the susceptibility to perceived stress. Based on these findings, neurobiologically-informed interventions with cognitive bias modification procedures should target the feeling of social disconnectedness to help students with alexithymic traits to better cope with psychosocial stress during transition phases. Furthermore, neurofeedback training targeting the insula might reduce the feeling of social isolation and therefore potentially enhance stress coping during stressful life events.

## Methods

### Subjects

Sixty healthy freshman students participated in the study after giving written informed consent. The study was approved by the institutional review board of the medical faculty of the University of Bonn and carried out in compliance with the latest revision of the Declaration of Helsinki. Subjects were screened prior to the first test session and received monetary compensation for study participation. Subjects had no past or present physical or psychiatric illness, as assessed by a medical history questionnaire and the Mini-International Neuropsychiatric Interview^42^. All subjects started their first semester without ever attending university courses before. Six subjects had to be excluded because they missed the second fMRI appointment (n = 3), showed excessive head motion in the MRI (> 3 mm/º; n = 2) or because of technical failures (n = 1). Therefore, final analyses include data from 54 healthy freshman (39 women, mean age: 18.85 ± 0.88 years minimum [min]/maximum [max] age: 18/22; alexithymia: 45.06 ± 8.79, min/max: 25/68; loneliness: 31.30 ± 5.44, min/max: 20/54). Four subjects missed one of their monthly appointments resulting in data loss of 1.48%.

### Experimental design

Subjects were monitored during their first semester at university for a total duration of 6 months, starting with a screening session in their first university month. Shortly (average: 14 days, min/max = 0/32 days) after the screening session, a first fMRI session was conducted (T1 = first month). The fMRI measurements were repeated after 6 months (T7 = seventh month; time between the two fMRI measurements = 164 days, min/max = 153/197 days). Participants completed several questionnaires every month between the two fMRI sessions measuring perceived stress, loneliness and social network size (see Fig. S1).

### Questionnaires

Subjects completed different sets of questionnaires to continuously monitor social behavior during their first semester. In the screening session and before the second fMRI scan, we assessed alexithymia (TAS [Toronto Alexithymia Scale]^43^), loneliness (UCLA LS [UCLA Loneliness Scale]^44^) and perceived stress (PSS-10 [Perceived Stress Scale]^45^). Furthermore, we monitored psychiatric symptoms during the transition phase by measuring social interaction anxiety (SIAS [Social Interaction Anxiety Scale]^46^), social anxiety (LSAS [Liebowitz Social Anxiety Scale]^47^), general trust (GTS [Yamagishi General Trust Scale]^48^), autistic-like traits (AQ [Autism Spectrum Quotient]^49^), depression symptoms (BDI [Becks Depression Inventory]^50^) and trait anxiety (STAI [State Trait Anxiety Inventory]^51^). Moreover, to differentiate between subjectively perceived social isolation (i.e. loneliness) and objective social network indices, we included the Social Network Size Questionnaire (SNS)^52^. We further assessed social support (F-SozU [Fragebogen zur Sozialen Unterstützung, short version K-14]^53^) as a key resilience factor during transition phases, to further distinguish between perceived social isolation and perceived social support. Every month between these sessions, subjects completed the PSS-10, UCLA LS and SNS. For a detailed description of the TAS, UCLA LS and PSS-10, see SI Methods.

### fMRI data acquisition

At the start of the experiment, subjects were instructed to lay as calm as possible. Functional data were acquired with a 3 T Siemens TRIO MRI system (Siemens AG, Erlangen, Germany) with a Siemens 32-channel head coil and obtained by using a T2*-weighted echoplanar (EPI) sequence [TR = 2690 ms, echo time (TE) = 30 ms, ascending slicing, matrix size: 96 × 96, voxel size: 2 × 2 × 3 mm^3^, slice thickness = 3.0 mm, distance factor = 10%, field of view (FoV) = 192 mm, flip angle 90°, 41 axial slices]. High-resolution T1-weighted structural images were collected on the same scanner (TR = 1660 ms, TE = 2.54 ms, matrix size: 256 × 256, voxel size: 0.8 × 0.8 × 0.8 mm^3^, slice thickness = 0.8 mm, FoV = 256 mm, flip angle = 9°, 208 sagittal slices). To control for inhomogeneity of the magnetic field, fieldmaps were obtained for each T2*-weighted EPI sequence [TR = 392 ms, TE (1) = 4.92, TE (2) = 7.38, matrix size: 64 × 64, voxel size: 3 × 3 × 3 mm^3^, slice thickness = 3.0 mm, distance factor = 10%, FoV = 192 mm, flip angle 60°, 37 axial slices].

### fMRI task

During the fMRI, subjects completed a well-established emotional face-matching paradigm^54,55^. To ensure the subjects’ attention, subjects had to match the identity of two simultaneously presented pictures at the bottom of the screen with a target picture presented at the top. Stimuli consisted of face pictures (neutral, fearful and happy) and houses as non-social control stimuli. Stimuli were presented with Presentation 14 software (Neurobehavioral Systems, Albany, CA, USA) in three blocks for every condition (Happy, Fearful, Neutral, House) with each block consisting of five trials. Stimuli did not vary in emotional expression or in sociality during a block. Trial duration was 5 s with a 10 s pause after each block. In this pause, a fixation-cross was depicted. The identity of the face stimuli varied between T1 and T7 to reduce habituation effects. Participants could choose their responses using an MRI-compatible response grip system (NordicNeuroLab AS, Bergen, Norway). Responses and reaction times (RTs) were measured to evaluate possible attention effects. High-resolution anatomical images were acquired after the functional images.

### fMRI analysis

The fMRI data were pre-processed and analyzed using standard procedures in SPM12 (Wellcome Trust Center for Neuroimaging, London, UK; http://www.fil.ion.ucl.ac.uk/spm) implemented in Matlab (The MathWorks Inc., Natick, MA). Participants with excessive head movements (> 3 mm/° in any direction, n = 2) or missing data due to technical failures (n = 1) were excluded from fMRI analyses. The first five volumes of each functional time series were discarded to allow for T1 equilibration. Functional images were corrected for head movements between scans by an affine registration. Images were initially realigned to the first image of the time series before being re-realigned to the mean of all images. To correct for signal distortion based on B0-field inhomogeneity, the images were unwarped by applying the voxel displacement map (VDM file) to the EPI time series (Realign & Unwarp). Normalization parameters were determined by segmentation and non-linear warping of the structural scan to reference tissue probability maps in MNI space. Normalization parameters were then applied to all functional images, which were resampled at 2 × 2 × 2 mm^3^ voxel size. For spatial smoothing, a 6-mm full width at half maximum Gaussian kernel was used. Raw time series were detrended using a high-pass filter (cut-off period 128 s).

A two-stage approach based on the general linear model implemented in SPM12 implemented in Matlab (The MathWorks Inc., Natick, MA, USA) was used for statistical analyses. On the first level, participants’ individual data were modelled using a fixed-effect model. Onsets and durations of the four experimental condition blocks (‘Happy, ‘Fearful’, ‘Neutral’, ‘House’) were modelled by a boxcar function convolved with a hemodynamic response function (HRF). Movement parameters were included in the design matrix as confounds. On the second-level, main contrasts of interest [Fearful _First_ > Neutral _First_; Happy _First_ > Neutral _First_; Fearful _Second_ > Neutral _Second_; Happy _Second_ > Neutral _Second_; Happy _First_ and Fearful _First_ > Neutral _First_; Happy _Second_ and Fearful _Second_ > Neutral _Second_; Happy _First > Second_ and Fearful _First > Second_ > Neutral _First > Second_; Happy _First & Second_ and Fearful _First & Second_ > Neutral _First & Second_] were computed using one sample t-tests. Loneliness and alexithymia ratings were used as covariates for the second level analysis. The following whole-brain analysis was done with a height threshold of *p* < 0.001. The main analyses of fMRI data focused on regions of interests (ROIs) associated with emotion processing in alexithymia and loneliness consisting of the amygdala, ACC and insular cortex^18^. These ROIs were anatomically defined according to the Wake Forest University PickAtlas (wfu PickAtlas) for both hemispheres. Parameter estimates of significant ROI clusters were extracted using MarsBaR (http://marsbar.sourceforge.net) and further analyzed in SPSS 25 (IBM Corp., Armonk, NY, USA).

### Statistical analyses

Repeated measures analyses of variance (ANOVAs) and Bonferroni corrected post-hoc *t*-tests were calculated using SPSS 25 (IBM Corp., Armonk, NY, USA) to examine changes in stress, loneliness and social network size over time. If the assumption of sphericity was significantly violated as assessed by Mauchly’s tests, Greenhouse Geisser corrections were applied. Pearson correlations between parameter estimates of significant ROI clusters, loneliness, perceived stress and alexithymia were calculated. Furthermore, mediation analyses were carried out using the PROCESS macro v3.4 for SPSS^56^. Focusing on mean stress as outcome variable, we used T1 alexithymia as predictor variable and mean loneliness ratings as mediator. As we were interested in the neurobiological mechanisms underlying the link between alexithymia, loneliness and perceived stress, we also tested the hypothesized mediation effects of the neural correlates of alexithymia and loneliness. Parameter estimates of significant clusters associated with alexithymia or loneliness at the first fMRI session were thus included as additional mediator variables and mediation effects were tested for each pathway between the former mentioned behavioral results. For all mediation analyses, 10,000 bootstraps samples were used.

## Supplementary Information

Supplementary Information 1.

## Data Availability

The data that support the findings of the present study are openly available in the repository of the Open Science Foundation at https://osf.io/csn5u/?view_only=21e52c0df9e14712894596967c4511bc.
