# Supplementary material for: Insula reactivity mediates subjective isolation stress in alexithymia
Source: Sci Rep. 2021 Jul 28;11:15326. doi: 10.1038/s41598-021-94799-w (PMC8319294; doi:10.1038/s41598-021-94799-w)
Supplement: Supplementary file 1 — Supplementary Information 1. [file 41598_2021_94799_MOESM1_ESM.docx]

**Supporting Information**

Insula reactivity mediates subjective isolation stress in alexithymia

Mitjan Morr^1,*,#^, Jana Lieberz^1,#^, Michael Dobbelstein^1^, Alexandra Philipsen^2^,

René Hurlemann^3,4^, Dirk Scheele^1,3,*^

* *Corresponding authors:*

Mitjan Morr

Division of Medical Psychology

University Hospital Bonn

Venusberg-Campus 1

53127 Bonn, Germany

Phone: +49 (0)228-287 19578

Fax: +49 (0)228 287 19125

E-mail: Mitjan.Morr@ukbonn.de

Dirk Scheele, Ph.D.

Department of Psychiatry
University of Oldenburg
Hermann-Ehlers-Str. 7
26160 Bad Zwischenahn, Germany
+49-(0)441-9615-1504

E-mail: Dirk-Scheele@gmx.de

**This PDF file includes:**

Supplementary Results

Tables S1 to S4

**Supplementary Methods**

**Detailed description of questionnaires**

The UCLA Loneliness Scale (UCLA LS) ^1^, the Toronto Alexithymia Scale (TAS) ^2^ and the Perceived Stress Scale (PSS-10) ^3^ were used for our main analyses. The UCLA LS is a widely used tool to measure loneliness in adolescents and adults. The questionnaire consists of 20 items with 10 positive and 10 negative framed statements. Subjects can respond on a scale from 1 “Never” to 4 “Often” and negative statements (which assess the opposite of loneliness) are recoded before calculating a sum score. The UCLA LS does not ask explicitly about loneliness to reduce social desirability. The validity and reliability are well established in different samples and languages ^4-7^. We observed moderate to excellent re-test reliabilities in our sample (all *p*s < 0.01; all *r*s > 0.63). Furthermore, we found adequate internal consistencies (Cronbach’s α between 0.72 and 0.83).

The Toronto Alexithymia Scale (TAS) is a 20-item self-report scale measuring alexithymic traits. In total, 5 items were recoded before calculating sum scores. The TAS consists of three subscales: difficulties describing feelings (DDF), difficulties identifying emotions (DIF) and externally orientated thinking (EOT). Responses are given on a 5-point scale ranging from 1 “strongly disagree” to 5 “strongly agree” with higher ratings indicating higher alexithymia. Reliability and validity for this questionnaire are well established ^8-10^. We observed a strong correlation between TAS scores at T1 and T7 (*r*_(52)_ = 0.67, *p* < 0.01) although TAS scores were significantly increased after six months. Cronbach’s α of 0.80 for the first TAS measurement and 0.82 for the second measurement indicate good internal consistency.

Finally, the Perceived Stress Scale (PSS-10) is a widely used questionnaire to measures global subjective stress. The questionnaire contains 10 short questions to determine stress levels in the last month. Subjects respond on a 5-point scale from 0 “never” to 4 “very often” and 4 items have to be recoded before a sum score is calculated. Various studies confirmed adequate reliability and validity of the PSS-10 ^11-13^. Again, we tested re-test reliability and found significant correlations between all measurement points (all *p*s < 0.01; all *r*s > 0.36) and good internal consistencies (Cronbach’s α between 0.82 and 0.87).

**Supplementary Results**

**Comparison of neuropsychological data between first (T1) and second functional magnetic resonance imaging (fMRI) session (T7)**

Subjects showed a significant increase in depression symptoms (*t*_(53)_ = 3.19, *p* < 0.01, *d* = 0.53), alexithymia (*t*_(53)_ = 2.83, *p* < 0.01, *d* = 0.32), social interaction anxiety (as measured by both Social Interaction Anxiety Scale: *t*_(53)_ = 3.05, *p* < 0.01, *d* = 0.26 and Liebowitz Social Anxiety Scale: *t*_(53)_ = 2.06, *p* = 0.05, *d* = 0.23) and performance anxiety (*t*_(53)_ = 2.50, *p* = 0.02, *d* = 0.25) after their first six months at the university (see **Table S2**). In contrast, neither loneliness (*t*_(53)_ = -0.98, *p* = 0.33, *d* = -0.12), general trust (*t*_(53)_ = 0.75, *p* = 0.46, *d* = 0.09) nor perceived social support (*t*_(53)_ = -0.80, *p* = 0.43, *d* = -0.10) changed significantly.

**Further correlation analyses of neuropsychological data**

Current perceived stress positively correlated with current alexithymia (T1: *r*_(52)_ = 0.49, *p* < 0.01; T7: *r*_(52)_ = 0.51, *p* < 0.01), depression symptoms (T1: *r*_(52)_ = 0.63, *p* < 0.01; T7: *r*_(52)_ = 0.72, *p* < 0.01) and social interaction anxiety (T1: *r*_(52)_ = 0.42, *p* < 0.01; T7: *r*_(52)_ = 0.50, *p* < 0.01) at the beginning and end of the observation period. Perceived stress also positively correlated with loneliness ratings at the end of the observation (T1: *r*_(52)_ = 0.24, *p* = 0.08; T7: *r*_(52)_ = 0.44, *p* < 0.01). In contrast, perceived stress did not correlate with social network size (T1: *r*_(52)_ = -0.06, *p* = 0.68; T7: *r*_(52)_ = -0.15, *p* = 0.29) or perceived social support (T1: *r*_(52)_ = -0.001, *p* = 0.99; T7: *r*_(52)_ = -0.18, *p* = 0.19; see **Table S3**).

**Behavioral functional imaging results**

The mean reaction times (RT), standard deviations and correct response rates (CR) of the emotional face-matching task are shown in **Table S4**. RTs did not significantly differ between the first and second fMRI session (Happy: *t*_(51)_ = 1.65, *p* = 0.11, *d* = 0.21; Fearful: *t*_(51)_ = 0.05, *p* = 0.96, *d* = 0.01; Neutral: *t*_(51)_ = 0.61, *p* = 0.54, *d* = 0.09; House: *t*_(51)_ = 0.33, *p* = 0.74, *d* = 0.05). Furthermore, the CRs did not differ between the sessions (Happy: *t*_(51)_ = 1.41, *p* = 0.16, *d* = 0.20; Fearful: *t*_(51)_ =1.07, *p* = 0.02, *d* = 0.01; Neutral: *t*_(51)_ = 0.47, *p* = 0.64, *d* = 0.07; House: *t*_(51)_ = 1.93, *p* = 0.06, *d* = 0.06). A repeated measure analysis of variance (ANOVA) with the factors time (T1 and T7) and stimulus type (happy, neutral, fearful faces and houses) revealed a significant main effect of stimulus type (*F*_(1.90,96.62)_= 9.98, *p* < 0.01, η_p_^2^ = 0.16). Post-Hoc paired sample t-tests revealed significantly smaller RTs in the fearful condition compared to happy (*t*_(52)_ = 3.53, *p*_cor_ < 0.01, *d* = 0.24), neutral (*t*_(52)_ = 2.83, *p*_cor_ = 0.04, *d* = 0.30) and house (*t*_(52)_ = 3.57, *p*_cor_ = 0.01, *d* = 0.24) stimuli at T1. RTs to fearful faces were also smaller at T7 in comparison with neutral (*t*_(52)_ = 2.91, *p*_cor_ = 0.03, *d* = 0.22) and house (*t*_(52)_ = 3.77, *p*_cor_ < 0.01, *d* = 0.48) stimuli. Furthermore, RTs to happy faces were faster at T7 than RTs to house stimuli (*t*_(52)_ = 2.87, *p*_cor_ = 0.04, *d* = 0.35). RTs did not correlate with extracted parameter estimates of any significant cluster (all *p*s > 0.5)

**Further** **correlation analyses of brain activation**

Exploratory whole-brain analyses confirmed that subjects with higher loneliness levels showed reduced activation in the right anterior cingulate cortex (ACC) in response to fearful faces compared to neutral ones in the first fMRI session (MNI_xyz_: 2, 26, 26, *k*_E_ = 135, *p*_FWE_ = 0.01 on cluster level). Furthermore, loneliness was associated with reduced left ACC activation in response to happy faces in contrast to neutral ones in the first fMRI session (MNI_xyz_: 0, 8, 26, *k*_E_ = 160, *p*_FWE_ = 0.01 on cluster level).

In the second fMRI session, higher loneliness was associated with stronger activation in the left caudate (coordinates of peak voxel in Montreal Neurological Institute space (MNI_xyz_): -14, 10, 22, *t*_(53)_ = 4.06, after familywise error corrections (*p*_FWE_) on cluster level *p*_FWE_ = 0.02) in response to fearful faces in contrast to neutral faces. No other significant associations with loneliness or alexithymia were observed for these contrasts on the whole brain level.

**TAS Subscale analysis**

Further mediation analyses were calculated by including the TAS subscales (“difficulties describing feelings” (DDF), “difficulties identifying feelings” (DIF), and “externally oriented thinking” (EOT)) as predictor variables, mean loneliness as mediator and mean perceived stress as outcome variable. Results again revealed a significant indirect effect of TAS DDF on stress via loneliness (β = 0.16, SE = 0.10, 95 % CI = 0.02 to 0.40)). Likewise, the effect of TAS DIF on perceived stress was partially mediated by loneliness (indirect effect β = 0.16, SE = 0.11, 95 % CI = 0.02 to 0.43; direct effect: (β = 0.37, *p* < 0.01, SE = 0.12, 95 % CI = 0.12 to 0.62). However, for TAS EOT, neither a direct (β = 0.04, *p* = 0.77, SE = 0.12, 95 % CI = -0.21 to 0.28) nor an indirect (β = -0.07, SE = 0.07, 95 % CI = -0.22 to 0.06) effect on stress was observed. TAS subscales were further included in our main analysis testing for serial mediation effects of anterior insula activity and loneliness (cf. **Fig. 3**). TAS DDF (serial mediation: β = 0.08, SE = 0.05, 95 % CI = 0.01 to 0.19) and DIF (serial mediation: β = 0.04, SE = 0.03, 95 % CI = 0.01 to 0.13) lead to a similar result as our main analysis using the total TAS score with reduced insula reactivity and loneliness mediating the link between alexithymia and stress. By contrast TAS EOT showed neither a direct (β = 0.07, *p* = 0.57, SE = 0.13, 95 % CI = -0.18 to 0.32) nor indirect effect (β = 0.06, SE = 0.05, 95 % CI = -0.02 to 0.18).

**Reaction to neutral faces**

To investigate whether the neural responsiveness to neutral faces was associated with alexithymia or loneliness, we extracted the parameter estimates for neutral faces of our significant right amygdala cluster associated with alexithymia and of the insula and ACC clusters associated with loneliness. Notably, the neural responsiveness to neutral faces did not significantly correlate with alexithymia or loneliness (*p*s > 0.05). However, as baseline parameter estimates do not include any within-subject comparison to control for individual differences in brain activity, this finding should be interpreted with caution. Thus, we conducted an additional SPM analysis for the Neutral _T1_ > House _T1_ contrast. Again, we did not find any significant associations of brain activity with alexithymia or loneliness, suggesting that alexithymia and loneliness are not associated with altered response to neutral faces.

**Collinearity analysis**

T1 alexithymia correlated with loneliness at every measurement point in this study (all *p*s < 0.05). Therefore, an additional regression analysis with mean perceived stress as dependent variable and mean loneliness, T1 alexithymia and parameter estimates of the right insula cluster associated with loneliness as independent variables was conducted to assess tolerance and variance inflation factors (VIF) of all independent variables. Collinearity diagnostics indicated only negligible collinearity between all variables (all tolerances > 0.69, all VIF < 1.45).

**Power analysis**

The sample size of this study was chosen to be similar to the largest previous studies in a meta-analysis about the neural correlates of alexithymia (n = 15 studies with 12 – 60 participants) ^14^. Furthermore, we used G*Power 3 ^15^ to conduct a post-hoc sensitivity power analysis for correlation analyses. Given our final sample size (n = 54, 39 females), we have 80% power to detect an effect sizes of |ρ| = 0.362 at a p-value of 0.05. This suggests that our sample size was sufficient to detect a medium effect size for the association between alexithymia and subjective stress. Importantly, previous studies consistently observed higher correlations between alexithymia and neural activity ^14^ indicating that our study was sufficiently powered to detect the hypothesized effects.

**Supplementary Tables**

**Table S1.** Psychometric assessments of stress, loneliness and network size ratings during the observation period.

|  | T1 | T2 | T3 | T4 | T5 | T6 | T7 |
| --- | --- | --- | --- | --- | --- | --- | --- |
|  |  |  |  |  |  |  |  |
| Perceived stress ^a^ | 12.43 (6.70) | 12.61 (6.89) | 14.11 (6.49) | 15.87 (7.55) | 15.91 (7.42) | 14.20 (7.86) | 13.06 (7.20) |
|  |  |  |  |  |  |  |  |
| Loneliness ^b^ | 31.94 (5.40) | 31.41 (5.86) | 30.59 (6.24) | 31.75 (6.75) | 31.31 (6.13) | 30.92 (5.80) | 31.24 (6.73) |
|  |  |  |  |  |  |  |  |
| Social network ^c^ |  |  |  |  |  |  |  |
| Size | 16.94 (7.02) | 17.26 (6.07) | 17.60 (6.66) | 17.51 (6.05) | 17.49 (7.41) | 17.45 (7.71) | 18.37 (7.50) |
| Roles | 4.94 (1.27) | 4.85 (1.12) | 5.02 (1.15) | 4.92 (1.21) | 5.04 (1.34) | 4.92 (1.23) | 5.13 (1.67) |
| Networks | 1.76 (1.12) | 1.74 (0.87) | 1.83 (0.91) | 1.74 (0.81) | 1.70 (1.05) | 1.82 (1.18) | 1.96 (1.12) |

***Notes:*** Numbers are means with standard deviations (in brackets). Neuropsychological and personality characteristics were assessed by the German adaptions of the

^a^ PSS-10 (Perceived Stress Scale),

^b^ UCLA LS (UCLA Loneliness Scale),

^c^ SNS (Social Network Size Questionnaire).

T1-T7 reflecting monthly measurements from study entry (T1) to the last fMRI session (T7).

**Table S2.** Psychometric assessments at baseline and after six months**.**

|  |  | T1 | |  | T7 | |  |  | |  |  |
| --- | --- | --- | --- | --- | --- | --- | --- | --- | --- | --- | --- |
|  |  | M | SD |  | M | SD |  | | *t* | *p* | *d* |
| Depressive symptoms ^a^ | | 3.69 | 3.20 |  | 6.10 | 5.49 |  | | 3.19 | 0.002** | 0.53 |
| Social support ^b^ | | 64.00 | 5.76 |  | 63.33 | 7.25 |  | | -0.80 | 0.430 | -0.10 |
| Autistic-like traits ^c^ | | 15.70 | 4.92 |  | 16.70 | 5.59 |  | | 2.05 | 0.045* | 0.19 |
| Liebowitz ^d^ |  | 21.96 | 17.74 |  | 24.57 | 18.14 |  | | 1.51 | 0.137 | 0.15 |
| Social interaction anxiety | | 7.09 | 5.17 |  | 8.35 | 5.96 |  | | 2.06 | 0.045* | 0.23 |
| Performance anxiety | | 5.87 | 5.64 |  | 7.37 | 6.36 |  | | 2.50 | 0.015* | 0.25 |
| Social interaction avoidance | | 4.44 | 4.48 |  | 4.26 | 4.88 |  | | -0.27 | 0.787 | -0.04 |
| Performance avoidance | | 4.56 | 4.32 |  | 4.59 | 4.94 |  | | 0.06 | 0.951 | 0.01 |
| Social interaction anxiety ^e^ | | 34.72 | 12.88 |  | 38.37 | 14.65 |  | | 3.05 | 0.004** | 0.26 |
| Social network ^f^ | |  |  |  |  |  |  | |  |  |  |
| Size |  | 16.94 | 7.02 |  | 18.37 | 7.49 |  | | 1.23 | 0.225 | 0.20 |
| Roles |  | 4.94 | 1.27 |  | 5.13 | 1.17 |  | | 1.37 | 0.176 | 0.15 |
| Networks |  | 1.76 | 1.12 |  | 1.96 | 1.11 |  | | 1.65 | 0.105 | 0.18 |
| Trait anxiety ^g^ | | 49.57 | 2.42 |  | 49.26 | 2.23 |  | | -0.77 | 0.444 | -0.14 |
| Alexithymia ^h^ |  | 43.5 | 8.50 |  | 46.61 | 10.72 |  | | 2.83 | <0.001** | 0.32 |
| Loneliness ^i^ |  | 31.94 | 5.40 |  | 31.24 | 6.73 |  | | -0.98 | 0.332 | -0.12 |
| General trust ^j^ | | 3.72 | 0.53 |  | 3.77 | 0.67 |  | | 0.75 | 0.459 | 0.09 |

***Notes:*** Neuropsychological and personality characteristics were assessed by the German adaptions of the

^a^ BDI (Becks Depression Inventory, Version II),

^b^ F-SozU (Fragebogen zur Sozialen Unterstützung, short version K-14),

^c^ AQ (Autism Spectrum Quotient),

^d^ LSAS (Liebowitz Social Anxiety Scale [Subscales SI = Social Interaction, P = Performance]),

^e^ SIAS (Social Interaction Anxiety Scale),

^f^ SNS (Social Network Size Questionnaire),

^g^ STAI (State Trait Anxiety Inventory),

^h^ TAS (Toronto Alexithymia Scale),

^i^ UCLA LS (UCLA Loneliness Scale),

^j^ GTS (Yamagishi General Trust Scale),

* *p* < 0.05, ** p < 0.01, M, mean, SD, standard deviation, T1, first measurement, T7, last measurement (after 6 months).

| **Table S3.** Correlations between perceived stress and psychometric assessments at the first and last measurement | | | |
| --- | --- | --- | --- |
|  |  | T1 | T7 |
| Loneliness |  | 0.24 | 0.44** |
| Alexithymia |  | 0.49** | 0.51** |
| Social networks | | -0.00 | -0.09 |
| Social roles |  | 0.00 | -0.10 |
| Social network size | | -0.06 | -0.15 |
| Social support | | -0.00 | -0.18 |
| Autistic-like traits |  | 0.09 | 0.33* |
| Depressive symptoms |  | 0.63** | 0.72** |
| Liebowitz social interaction anxiety |  | 0.41** | 0.47** |
| Social interaction anxiety |  | 0.42** | 0.43** |
| Trait anxiety |  | -0.06 | -0.07 |

***Notes:*** * *p* < 0.05, ** *p* < 0.01, T1, first measurement, T7, last measurement (after 6 months).

**Table S4.** Mean and standard deviation of response times (in s) and correct responses rates in the fMRI task

|  | M | | SD | | CR (%) | | SD (%) | |
| --- | --- | --- | --- | --- | --- | --- | --- | --- |
|  | T1 | T7 | T1 | T7 | T1 | T7 | T1 | T7 |
| Fearful | 1.26 | 1.25 | 0.26 | 0.21 | 99.11 | 98.74 | 2.28 | 2.94 |
| Happy | 1.33 | 1.27 | 0.32 | 0.26 | 98.49 | 97.73 | 3.37 | 4.12 |
| Neutral | 1.32 | 1.30 | 0.30 | 0.25 | 98.74 | 98.61 | 2.63 | 3.03 |
| House | 1.37 | 1.36 | 0.24 | 0.25 | 99.11 | 97.98 | 2.63 | 3.36 |

***Notes:*** M, mean, CR, correct response rate, SD, standard deviation, T1, first measurement, T7, last measurement (after 6 months).

**Supplementary figures**


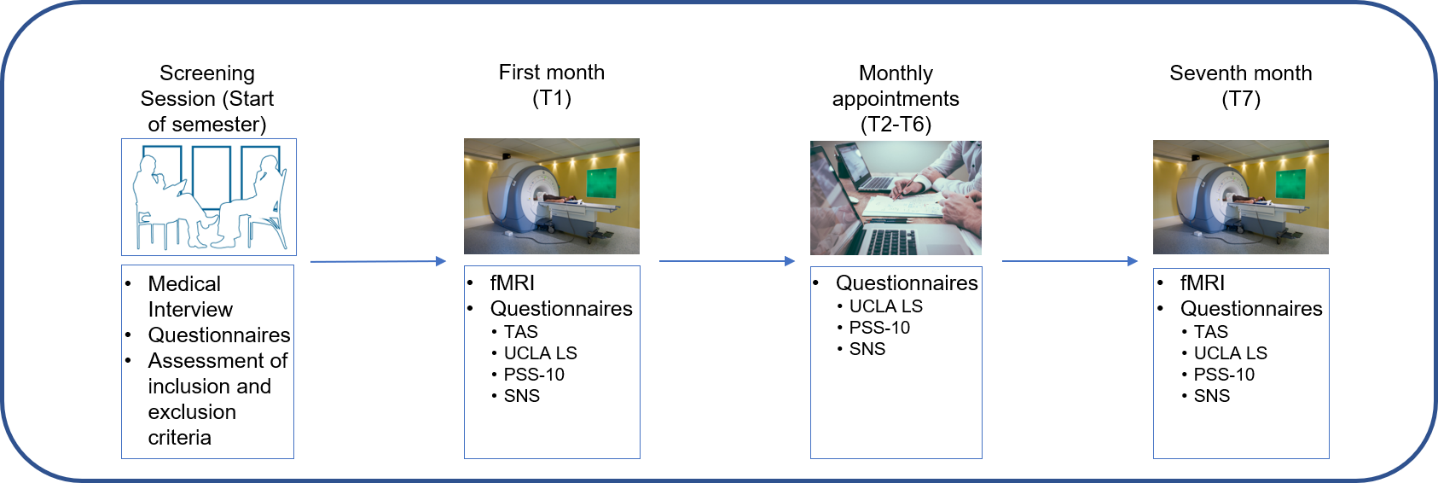


**Figure S1.**

This study started with a screening session consisting of a medical interview and questionaires. All subjects started their first semester without ever attending university courses before. Shortly (average: 14 days, min = 0, max = 32) after the screening session, a first fMRI session was conducted (T1 = first month). The fMRI measurements were repeated after six months (T7 = seventh month; time between the two fMRI measurements = 164 days, min = 153, max = 197). In the 5 months between the two fMRI sessions, loneliness (UCLA LS), psychosocial stress (PSS-10) and social network size (SNS) was measured via questionnaires (T2-T6). License free stock photos derived from pixabay.com.

**Supplementary References**

1 Russell, D., Peplau, L. A., & Cutrona, C. E. The revised UCLA Loneliness Scale: concurrent and discriminant validity evidence. *J Pers Soc Psychol* **39**, 472-480 (1980).

2 Taylor, G. J., Ryan, D., & Bagby, R. M. Toward the development of a new self-report alexithymia scale. *Psychother Psychosom* **44**, 191-199 (1985).

3 Cohen, S., Kamarck, T., & Mermelstein, R. A global measure of perceived stress. *J Health Soc Behav* **24**, 385-396 (1983).

4 Döring, N. & Bortz, J. Psychometrische Einsamkeitsforschung: Deutsche Neukonstruktion der UCLA Loneliness Scale. [Psychometric research on loneliness: A new German version of the University of California at Los Angeles (UCLA) Loneliness Scale.]. *Diagnostica* **39**, 224-239 (1993).

5 Russell, D. W. UCLA Loneliness Scale (Version 3): reliability, validity, and factor structure. *J Pers Assess* **66**, 20-40 (1996).

6 Vassar, M. & Crosby, J. W. A reliability generalization study of coefficient alpha for the UCLA Loneliness Scale. *J Pers Assess* **90**, 601-607 (2008).

7 Lasgaard, M. Reliability and validity of the Danish version of the UCLA Loneliness Scale. *Pers Individ Differ* **42**, 1359-1366 (2007).

8 Bagby, M., Taylor, G. J., & Parker, J. D. Construct validity of the Toronto alexithymia scale. *Psychother Psychosom* **50**, 29-34 (1988).

9 Bagby, R. M., Taylor, G. J., & Parker, J. D. The twenty-item Toronto Alexithymia Scale—II. Convergent, discriminant, and concurrent validity. *J Psychosom Res* **38**, 33-40 (1994).

10 Parker, J. D., Taylor, G. J., & Bagby, R. M. The 20-Item Toronto Alexithymia Scale: III. Reliability and factorial validity in a community population. *J Psychosom Res* **55**, 269-275 (2003).

11 Reis, D., Lehr, D., Heber, E., & Ebert, D. D. The German Version of the Perceived Stress Scale (PSS-10): Evaluation of Dimensionality, Validity, and Measurement Invariance With Exploratory and Confirmatory Bifactor Modeling. *Assessment* **26**, 1246-1259 (2017).

12 Klein, E. M. *et al.* The German version of the Perceived Stress Scale - psychometric characteristics in a representative German community sample. *BMC Psychiatry* **16**, 159 (2016).

13 Baik, S. H. *et al.* Reliability and validity of the Perceived Stress Scale-10 in Hispanic Americans with English or Spanish language preference. *J Health Psychol* **24**, 628-639 (2019).

14 van der Velde, J. *et al.* Neural correlates of alexithymia: a meta-analysis of emotion processing studies. *Neurosci Biobehav Rev* **37**, 1774-1785 (2013).

15 Faul, F., Erdfelder, E., Lang, A.-G. & Buchner, A. G* Power 3: A flexible statistical power analysis program for the social, behavioral, and biomedical sciences. *Behav res methods* **39**, 175-191 (2007).
